# Annual PM_2.5_ and cardiovascular mortality rate data: Trends modified by county socioeconomic status in 2,132 US counties

**DOI:** 10.1016/j.dib.2020.105318

**Published:** 2020-03-16

**Authors:** Lauren H. Wyatt, Geoffrey Colin L. Peterson, Tim J. Wade, Lucas M. Neas, Ana G. Rappold

**Affiliations:** a United States Environmental Protection Agency, Research Triangle Park, NC 27709, United States; b ORISE at National Health and Environmental Effects Research Laboratory/Environmental Public Health Division, United States Environmental Protection Agency, Research Triangle Park, NC, 27709, United States

**Keywords:** Mortality, Cardiovascular, Air pollution, Particulate matter, Socioeconomic status

## Abstract

This article contains data on county-level socioeconomic status for 2132 US counties and each county's average annual cardiovascular mortality rate (CMR) and fine particulate matter (PM_2.5_) concentration for 21 years (1990–2010). County CMR, PM_2.5_, and socioeconomic data were obtained from the US National Center for Health Statistics, US Environmental Protection Agency's Community Multiscale Air Quality modeling system, and the US Census, respectively. Annual socioeconomic indices were created using seven county-level measures from the 1990, 2000, and 2010 US Census using factor analysis. Quintiles of this index were used to generate categories of county socioeconomic status. This national data set contains data for annual PM_2.5_ and CMR changes over a time-period when there was a significant reduction in US air pollutants (following the enactment of the 1970 Clean Air Act). These data are associated with the article “The contribution of improved air quality to reduced cardiovascular mortality: Declines in socioeconomic differences over time” [1]. Data are stored in a comma separated value format and can be downloaded from the USEPA ScienceHub data repository (https://doi.org/10.23719/1506014).

**Table:** Specifications Table

Subject	Public Health and Health Policy
Specific subject area	Air pollution, cardiovascular mortality, social epidemiology
Type of data	Table
How data were acquired	Annual mortality and PM_2.5_ data were obtained from the US National Center for Health Statistics (NCHS) and Community Multiscale Air Quality (CMAQ) modeling system, respectively. County socioeconomic variables were acquired from the U.S. Census (years 1990, 2000, and 2010), county population size from the 2000 census, and healthcare variables from the US Census's County Business Patterns. County socioeconomic indexes for the years 1990, 2000, and 2010 were created with maximum-likelihood factor analysis of socioeconomic factors seven factors. Densities of health care facilities in each county were estimated using North American Industry Classification System (NAICS) codes. Data aggregation and analysis was performed using the software R (version 3.6.1).
Data format	Raw Analyzed
Parameters for data collection	Counties included in this dataset were located in the contiguous US and had a population of at least 20,000 in every year, to comply with NCHS data privacy standards.
Data source location	2132 counties in the United States. Unique county identifiers are provided in each table using codes that include state and county identifiers (Federal Information Processing Standard, FIPS).
Data accessibility	Repository name: EPA ScienceHub Direct URL to data: https://doi.org/10.23719/1506014
Related research article	Wyatt Lauren H., Geoffrey Colin L., Wade Tim J., Neas Lucas M., Rappold Ana G., The contribution of improved air quality to reduced cardiovascular mortality: Declines in socioeconomic differences over time, Environment International 136: 105430 DOI: https://doi.org/10.1016/j.envint.2019.105430

**Table:** 

**Value of the Data** • This dataset combines national data from health, environmental, and social domains over a time-period when great reductions in PM_2.5_ were observed (1990–2010). • The dataset is a valuable resource for researchers interested in measuring PM_2.5_-related trends in CMR with respect to county-level factors. • Methods in creating the SES and healthcare indices can be helpful in constructing similar indices.

## 1 Data

This dataset contains 1 data dictionary and 6 data files that describe annual county cardiovascular mortality rates (CMR) and fine particulate matter (PM_2.5_) concentrations for 21 years (1990–2010) and county-level factors including socioeconomic factors, socioeconomic status (SES) index, density of healthcare establishments, and population characteristics. Data files are available online through the EPA ScienceHub [https://doi.org/10.23719/1506014]:

• County_annual_PM25_CMR.csv has the annual average PM_2.5_ (μg/m^3^) and cardiovascular mortality rate (deaths/100,000 person-years) for each county for each year between 1990 and 2010.
• County_RAW_variables.csv includes data on county factors including socioeconomic, population, and healthcare variables for each county. Socioeconomic variables for the years 1990, 2000, and 2010 included civilian unemployment rates, median household (HH) income, percent female HHs with no spouse, percent of owner occupied housing units, percent of individuals aged 25 and older that graduated high school or had greater education, and percent of households and families below poverty. Population variable included county population size from the 2000 Census. Healthcare facility variables included the density of facilities for the years 1999 and 2005 which were identified from the US Census's County Business Patterns (CBP) files (cbp99co.txt and cbp05co.txt) based on the North American Industry Classification System (NAICS) codes listed in the file “Healthcare_NAICS2007.csv”.
• County_SES_index_quintile.csv contains data on the SES indices for 1990, 2000, and 2010 created using a factor analysis of Census data from County_RAW_variables.csv. Lower index values denote lower socioeconomic deprivation (i.e. counties with fewer HHs below poverty, greater HH income, greater educational attainment, less unemployment, greater educational attainment).
• Healthcare_NAICS2007.csv is a list of the NAICS codes for facilities identified as healthcare establishments, previously used to create the EPA's Environmental Quality Index [2].
• cbp99co.txt is a raw data file from the 1999 US Census's County Business Patterns (CBP) containing the number of establishments, employment during the week of March 12, first quarter payroll, and annual payroll by county, with respect to the designated NAICS code.
• cbp05co.txt is a raw data file from the 2005 CBP containing the number of establishments, employment during the week of March 12, first quarter payroll, and annual payroll by county, with respect to the designated NAICS code.
• Data_dictionary.xlxs contains the data dictionary with the list of variables, variable descriptions, and data sources where applicable for each.csv file.

Differences between SES quintiles with respect to the three 1990 census variables that contributed the most to the index (percent high school graduate or greater education (age 25+), median household income, and percent households below poverty) are shown in Figure S2 of the related research article. Table S1 in that article describes the SES factor loadings that created the 1990 SES index. County SES classifications using the 1990 Census data for the 2132 counties included in the dataset are depicted in Figure S3 of the related research article. Trends in the cardiovascular mortality rate and PM_2.5_ between 1990 and 2010 nationally and by SES quintile are depicted in Figure 1 of the related research article [1].

## 2 Experimental design, materials, and methods

### 2.1 County-level socioeconomic, population, and healthcare related characteristics

County socioeconomic variables acquired from the 1990, 2000, and 2010 US Census included seven socioeconomic variables: percent households below poverty, median household income, percent high school graduate or greater education (percent of individuals aged 25 and older that graduated high school or had greater education), civilian unemployment rate, percent female households with no spouse, percent vacant housing units, and percent owner occupied housing units [[3], [4], [5]]. These variables were used to calculate county SES indices by performing maximum-likelihood factor analysis with a standard rotation (varimax). Loadings from the first factor (∼40.5% of the total variance) were used to calculate SES scores. The SES index loadings for the baseline year (1990) are shown in Table S1 of the related research article [1]. Quintiles of scores were used to define SES strata: Q1, highest SES; Q2, high SES; Q3, medium SES; Q4, low SES; and Q5, lowest SES.

Data on county population was obtained from the 2000 Census.

Data on county healthcare facilities were determined using information from the US Census's County Business Patterns. The density of health care facilities (facilities per 1000 individuals) in each county was estimated using NAICS codes to identify healthcare establishments. Potential healthcare related codes were identified by using the same codes used in creating the healthcare component of the EPA's Environmental Quality Index [2].

### 2.2 Mortality data

Annual age-adjusted cardiovascular mortality rates (CMR) were calculated from individual-level records from the nationally inclusive dataset, recorded by the U.S. National Center for Health Statistics (NCHS), for each year between 1990 and 2010, standardized to the National Cancer Institute's 2000 standard population [6], and expressed as the number of deaths/100,000 person-years. Cardiovascular mortality was identified using cause of death codes (International Classification of Diseases, Ninth Revision, 390–434 and 436–448). We used data for 2132 counties across the contiguous U.S. with a population of at least 20,000, to comply with NCHS data privacy standards.

### 2.3 Air quality data

Average annual fine particulate matter (PM_2.5_) concentrations were estimated using the Community Multiscale Air Quality (CMAQ) modeling system [7] that predicts concentration of airborne gases and particles using a deterministic model of atmospheric chemistry and transport from anthropogenic and non-anthropogenic sources. Air quality was simulated over the whole period on a 36 km-grid using internally consistent historic emissions with lateral boundary conditions derived from the hemispheric simulations. Annual concentrations of PM_2.5_ were calculated using the daily averages of hourly concentrations. CMAQ-estimated PM_2.5_ concentrations were first interpolated to census block population centroids and then aggregated into county population-weighted concentrations based on Census 2000 population data.

## Disclaimer

The views expressed in this manuscript are those of the individual authors and do not necessarily reflect the views and policies of the U.S. Environmental Protection Agency. Mention of trade names or commercial products does not constitute endorsement or recommendation for use.
